# Supplementary material for: Predicting visual function by interpreting a neuronal wiring diagram
Source: Nature. 2024 Oct 2;634(8032):113–23. doi: 10.1038/s41586-024-07953-5 (PMC11446822; doi:10.1038/s41586-024-07953-5)
Supplement: Supplementary file 6 — CRF and ERF predictions for individual TmY4 and TmY9 cells. Analogous to Supplementary Data 3, but for TmY target types. Shown are the top four monosynaptic pathways, the strongest pathway passing through each of the top ten intermediary types (ranking from Extended Data Fig. 7), and the trisynaptic pathway Tm1–TmY–Dm3–TmY (see the section entitled Prediction of spatial normalization). [file 41586_2024_7953_MOESM6_ESM.zip › DataS4/TmY9qΓèÑ/720575940627532933.pdf]

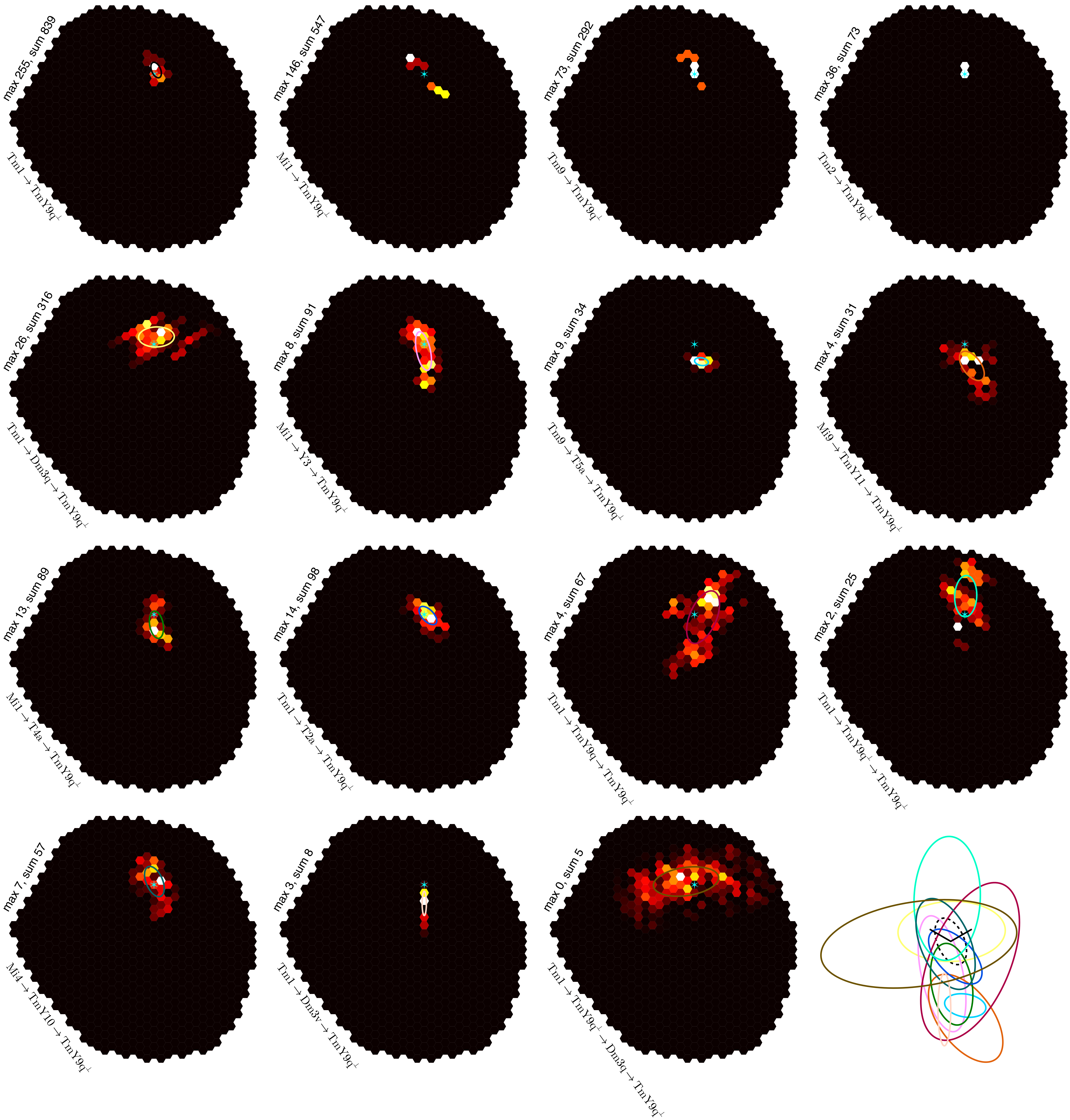
max 255, sum 839
Tm1 → TmY9q⊥
max 146, sum 547
Mi1 → TmY9q⊥
max 73, sum 292
Tm9 → TmY9q⊥
max 36, sum 73
Tm2 → TmY9q⊥
max 26, sum 316
Tm1 → Dm3q → TmY9q⊥
max 8, sum 91
Mi1 → Y3 → TmY9q⊥
max 9, sum 34
Tm9 → T5a → TmY9q⊥
max 4, sum 31
Mi9 → TmY11 → TmY9q⊥
max 13, sum 89
Mi1 → T4a → TmY9q⊥
max 14, sum 98
Tm1 → T2a → TmY9q⊥
max 4, sum 67
Tm1 → TmY9q⊥ → TmY9q⊥
max 2, sum 25
Tm1 → TmY9q⊥ → TmY9q⊥
max 7, sum 57
Mi4 → TmY10 → TmY9q⊥
max 3, sum 8
Tm1 → Dm3v → TmY9q⊥
max 0, sum 5
Tm1 → TmY9q⊥ → Dm3q → TmY9q⊥
